# The impact of empathy with nature on tourists’ responsible behavioral intentions in natural heritage tourism: the moderating roles of escapism and awe

**DOI:** 10.3389/fpsyg.2025.1661381

**Published:** 2026-01-05

**Authors:** Kang Wang, Shuangjun Huang, Zhen Chen, Yidong Liu

**Affiliations:** 1 School of Industrial Design, Hubei Institute of Fine Arts, Wuhan, China; 2 Graduate School, Hanyang University, Seoul, Republic of Korea

**Keywords:** awe, empathy with nature, escapism, natural heritage tourism, responsible behavioral

## Abstract

**Introduction:**

Natural heritage tourism provides opportunities for visitors to engage with ecological and cultural resources, yet encouraging responsible tourist behavior remains a persistent challenge for sustainable destination management. This study examines how tourists’ perceptions of natural and cultural environments shape their intentions to behave responsibly, with a particular focus on the mediating role of empathy with nature and the moderating influences of perceived escapism and awe.

**Methods:**

Using Shennongjia in Hubei Province, China, as the study site, data were collected through structured questionnaires administered to tourists. Structural equation modeling (SEM) was employed to analyze the relationships among environmental perceptions, empathy with nature, responsible behavioral intentions, and the effects of escapism and awe.

**Results:**

Findings show that both natural and cultural environmental perceptions significantly enhance empathy with nature, which in turn positively predicts tourists’ economic and non-economic responsibility behaviors. Empathy with nature mediates the effects of both environmental perceptions on responsible behaviors. Perceived escapism moderates the relationship between natural environmental perceptions and empathy, whereas awe strengthens the association between empathy and non-economic responsibility behaviors. However, escapism does not significantly moderate the influence of cultural perceptions, nor does awe significantly moderate the link between empathy and economic responsibility behaviors.

**Discussion:**

This study clarifies the dual roles of natural and cultural stimuli in shaping empathy-driven responsible behavior among tourists. The differentiated moderating effects of escapism and awe reveal nuanced boundary conditions for promoting responsible actions. Practically, the results highlight the importance for destination managers to integrate natural and cultural experiences, cultivate visitors’ empathy with nature, and strategically evoke escapism and awe to strengthen both economic and normative forms of sustainable tourist behavior.

## 1 Introduction

Natural heritage tourism has emerged as one of the most significant segments of global tourism, attracting visitors to sites that combine ecological richness with cultural and historical value. These destinations, encompassing national parks, nature reserves, and sites of combined natural–cultural significance, offer unique opportunities for individuals to experience landscapes, wildlife, and cultural narratives that are often unavailable in urban or artificial settings (Zhang Z. et al., 2023; Tian et al., 2023). Beyond recreational enjoyment, natural heritage tourism plays a crucial role in promoting environmental conservation, local economic development, and the transmission of cultural knowledge. However, achieving sustainable outcomes depends not only on environmental management but also on tourists’ responsible behavioral intentions, including both economic support for conservation and adherence to normative ecological practices (Lin et al., 2022; Gao et al., 2017).

Recent research highlights the importance of affective and cognitive engagement with natural and cultural environments in shaping tourists’ pro-environmental and responsible behaviors. In particular, empathy with nature—an individual’s capacity to emotionally resonate with and morally consider the natural environment—has been identified as a key psychological mechanism translating environmental perceptions into concrete actions (Berenguer, 2007; Tam, 2013; Di Fabio and Kenny, 2021). While prior studies have examined the general influence of connectedness to nature on pro-environmental behaviors, less attention has been paid to the interplay between natural and cultural stimuli, empathy, and differentiated behavioral outcomes, such as economic and non-economic responsibility behaviors (Mackay and Schmitt, 2019; Wang et al., 2023). Furthermore, emerging evidence suggests that affective states, including perceived escapism and awe, may moderate the strength of these relationships by enhancing tourists’ immersion, moral elevation, and cognitive receptivity (Hirschman, 1983; Keltner and Haidt, 2003; Piff et al., 2015).

Against this backdrop, the present study investigates the mechanisms through which tourists’ perceptions of natural and cultural environments in a natural heritage site influence their responsible behavioral intentions, with a focus on the mediating role of empathy with nature and the moderating effects of perceived escapism and awe. Using Shennongjia in Hubei Province, China, as a case study, this research applies a structural equation modeling approach to empirically test the proposed conceptual framework. By integrating environmental psychology, sustainable tourism, and heritage management perspectives, the study aims to provide both theoretical insights and practical guidance for enhancing responsible behaviors among visitors, thereby supporting the sustainable development of natural heritage destinations.

The research contributes to the literature in three primary ways. First, it clarifies the dual role of natural and cultural environmental perceptions in stimulating empathy with nature. Second, it examines the differentiated impact of empathy on economic versus non-economic responsibility behaviors, highlighting the psychological mechanisms that drive sustainable actions. Third, it explores the moderating roles of escapism and awe, offering insights into how affective states shape the translation of empathic engagement into behavioral intentions. Collectively, these contributions provide a comprehensive understanding of the emotional, cognitive, and motivational processes underlying tourists’ responsible behaviors in natural heritage tourism contexts.

Specifically, this study addresses the following research questions:

(1) In what ways are tourists’ perceptions of the natural and cultural environment associated with their empathy with nature in the context of natural heritage tourism?

(2) How does empathy with nature affect tourists’ economic and non-economic responsibility behaviors?

(3) Does empathy with nature mediate the relationship between environmental perceptions and responsible behavioral intentions?

(4) How do perceived escapism and awe moderate the relationships between environmental perceptions, empathy with nature, and tourists’ responsible behaviors?

By integrating environmental psychology, sustainable tourism, and heritage management perspectives, the study aims to provide both theoretical insights and practical guidance for enhancing responsible behaviors among visitors, thereby supporting the sustainable development of natural heritage destinations.

## 2 Literature review

### 2.1 Responsible behavioral intentions in natural heritage tourism

#### 2.1.1 Definition and scope of natural heritage tourism

Natural heritage tourism refers to travel and recreational activities that take place in areas recognized for their outstanding natural value, ecological integrity, and environmental significance. It encompasses both the appreciation of natural landscapes and the pursuit of conservation-compatible experiences that foster ecological awareness and sustainable development (Zhang Z. et al., 2023; Zhang and Lee, 2022). According to Zhang Z. et al. (2023), natural heritage tourism is an essential component of world heritage conservation, integrating the dual objectives of heritage preservation and visitor engagement. Their review emphasizes that natural heritage tourism is not simply tourism occurring within protected areas; rather, it is a value-driven form of nature-based tourism that aligns ecological conservation, cultural interpretation, and community participation under the broader goal of sustainability.

Building on this foundation, Adie et al. (2017) highlight that world heritage sites—whether cultural or natural—operate as powerful “place brands” that attract tourists through the perceived authenticity, prestige, and educational value associated with the World Heritage label. This symbolic dimension broadens the scope of natural heritage tourism beyond mere nature appreciation to include learning, identity formation, and global cultural recognition. In parallel, Engelhardt (2005) argues that the sustainable development of heritage tourism depends on local community management and the integration of ecological and cultural values, noting that cultural and natural heritages are often intertwined. This view is particularly relevant to sites such as Shennongjia in China, where biodiversity conservation coexists with traditional ecological knowledge and cultural heritage narratives.

From a sustainability perspective, Lee and Hsieh (2016) propose a set of ecological indicators for tourism management in natural wetland areas, emphasizing environmental quality, biodiversity, and carrying capacity as crucial components of sustainable tourism assessment. Similarly, Zolfani et al. (2015) identify natural heritage tourism as a subset of sustainable tourism, distinguished by its emphasis on environmental stewardship, intergenerational equity, and community wellbeing. These studies collectively indicate that natural heritage tourism cannot be understood solely through the lens of economic or recreational motivations; rather, it must be conceptualized as a multidimensional system that integrates ecological protection, cultural continuity, and social participation.

Further extending this perspective, Stronza and Gordillo (2008) and Genet and Kebede (2022) highlight the indispensable role of local communities in shaping sustainable tourism outcomes. Community perceptions, participation, and benefit-sharing mechanisms are central to ensuring that natural heritage tourism contributes to both conservation and livelihood improvement. In regions where cultural and natural values are inseparable—such as monastic landscapes or indigenous territories—heritage tourism often serves as a medium through which ecological, cultural, and spiritual dimensions intersect.

Taken together, the literature converges on the understanding that natural heritage tourism lies at the intersection of nature-based, heritage-oriented, and sustainability-focused tourism. It is characterized by the dual mandate of protecting ecological integrity and delivering meaningful visitor experiences that enhance environmental awareness and community empowerment. Thus, the scope of natural heritage tourism extends beyond passive sightseeing to include active engagement with conservation values, cultural interpretation, and responsible management practices that align with the principles of sustainable development.

#### 2.1.2 Conceptualization of responsible behavioral intentions (ERB and NERB)

Responsible tourism—as articulated in foundational policy documents such as the Cape Town Declaration (2002)—positions responsibility as a multi-dimensional construct encompassing environmental protection, social equity, and economic fairness in destination contexts. From a behavioral perspective, “responsible behavioral intentions” refer to tourists’ self-reported readiness to perform actions that support these normative goals while visiting a destination. Empirically and conceptually, it is useful to distinguish between economically-oriented responsible behaviors (ERB) and non-economic responsible behaviors (NERB). ERB typically denotes actions that involve a monetary or market facet—such as paying conservation fees, purchasing locally-produced sustainable goods, contributing to conservation donations, or choosing higher-priced but certified sustainable services—where tourists’ economic choices directly support conservation and local livelihoods. NERB, by contrast, denotes non-monetary, often normative or pro-social behaviors, including adherence to site rules, not littering, staying on designated trails, respectful conduct toward local cultures, and volunteering time or effort for conservation activities. This bifurcation helps capture distinct motivational antecedents and policy levers—market-based incentives and pricing strategies may primarily target ERB, whereas social norms, interpretation and on-site management primarily shape NERB (Cape Town Declaration, 2002).

Theoretical accounts explaining why tourists form ERB and NERB intentions draw on complementary strands. Gao et al. (2017) apply norm-activation theory (NAT) to show that awareness of consequences and personal norms predict tourists’ sense of responsibility, which in turn fosters behavioral intentions—mechanisms that map closely onto NERB outcomes where moral obligation and perceived normative salience are decisive. Social-interaction processes also matter: Lin et al. (2022) demonstrate that tourist-to-tourist interactions can activate normative cues and reputational concerns that increase compliance with site norms (NERB) and can even nudge market behaviors when peers signal support for local products (ERB). At a broader social-psychological level, the Social Identity Model of Pro-Environmental Action (SIMPEA) (Fritsche et al., 2018) highlights how group identification and collective efficacy shape pro-environmental intentions—an explanation particularly relevant for NERB that are publicly visible and socially sanctioned. Organizational and institutional logics further complement individual-level theories: Van Aaken et al. (2013) show that reputation and field-level norms motivate corporate pro-social engagement, suggesting analogous mechanisms at the destination/system level where institutional framing (e.g., destination certification, visible conservation investments) can facilitate ERB by reducing perceived transaction costs and reinforcing trust.

Empirical studies of community-oriented and sustainable tourism also illuminate the ERB/NERB distinction. Manyara and Jones (2007) document how community-based enterprises transform tourists’ economic choices into local benefits, underscoring how ERB (e.g., purchasing local services/products) translates into tangible livelihood outcomes. Conversely, research on visitor behavior in protected and heritage sites repeatedly finds that NERB—compliance with rules, low-impact conduct, and respect for cultural protocols—is more strongly predicted by interpretive interventions, on-site social norms, and perceived behavioral control than by price- or market-based incentives alone.

Psychological mechanisms that link pro-environmental feelings to responsibility intentions further bridge ERB and NERB literatures. Empathy and connectedness to nature research (e.g., Berenguer, 2007; Schultz, 2000) indicates that affective ties and perspective-taking toward nature increase moral concern and motivate both monetary support and non-monetary protective acts. Measurement work on connectedness (Mayer and Frantz, 2004) and experimental studies of anthropomorphism (Tam et al., 2013) show that strengthening perceived relationship with the natural world can raise both ERB (willingness to pay, donations) and NERB (rule compliance, caretaking behaviors). Meta-analytic evidence (Mackay and Schmitt, 2019) confirms a positive association between nature-connection constructs and protective behavior, though effect sizes vary by outcome type—often larger for deliberative, private behaviors (some forms of ERB) than for immediate, observed public behaviors (certain NERB), depending on contextual cues and social monitoring. Recent work also links personality and dispositional empathy to sustained pro-environmental intentions, indicating individual differences may moderate how affective connections translate into ERB vs. NERB (Di Fabio and Kenny, 2021).

Taken together, the literature suggests three pragmatic implications for empirical modeling. First, ERB and NERB should be operationalized and measured as distinct but related constructs, with item pools reflecting monetary/market actions versus normative/compliance actions. Second, explanatory models should combine moral-norm theories (e.g., NAT, VBN) and social-identity frameworks to capture drivers of NERB, while including institutional and market framing variables to capture ERB. Third, affective constructs—empathy with nature, connectedness—serve as psychological conduits that can motivate both ERB and NERB but may interact with situational moderators (social visibility, transaction friction, interpretive messaging) to determine which type of responsible behavior is more likely in a given context. These insights justify the present study’s separation of ERB and NERB as distinct dependent variables and motivate the inclusion of empathy with nature as a central mediating mechanism linking environmental perceptions to heterogeneous responsibility outcomes.

### 2.2 Environmental perceptions and empathy with nature

Empathy with nature is originally conceptualized as a relatively stable dispositional tendency that reflects individuals’ affective and cognitive responsiveness toward the natural world (Tam, 2013). Nonetheless, dispositional constructs may exhibit situational activation when individuals encounter evocative environmental stimuli in tourism settings (Kirillova et al., 2017). In natural heritage tourism, the natural environment provides sensory and aesthetic cues that can momentarily strengthen emotional connectedness to nature (Ballew and Omoto, 2018), while the cultural environment offers symbolic meanings and interpretive narratives that facilitate reflective and value-laden emotional responses (Zhou et al., 2025). Accordingly, this study examines how visitors’ perceptions of natural and cultural environments are associated with the situational activation of empathy with nature during the tourism experience, rather than assuming its causal formation. This conceptual clarification aligns the model with the dispositional nature of empathy with nature while allowing its contextual expression to be explored.

#### 2.2.1 Natural environment and empathy with nature

A growing body of literature emphasizes that the natural environment (NE)—including landscape aesthetics, biodiversity, ecological integrity, and sensory immersion—plays a central role in stimulating empathy with nature (EN). Butler (2000) proposed that direct encounters with authentic and pristine environments evoke affective responses such as appreciation, tranquility, and emotional attachment, which underlie an empathetic stance toward the natural world. Empirical studies further demonstrate that the perceived quality of natural landscapes positively shapes visitors’ emotional engagement. For instance, Chung et al. (2018) found that biodiversity and ecological richness not only enhance tourism attractiveness but also foster affective responses and moral concern for environmental conservation. Similarly, Cheng and Monroe (2012) revealed that emotional experiences derived from natural interaction—such as exploration, sensory contact, and aesthetic appreciation—promote affective connection and compassion toward nature, forming the basis of empathy.

Psychological research on nature connectedness reinforces this environmental pathway. Tam (2013) conceptualized dispositional empathy with nature as a stable emotional capacity that is activated through exposure to natural cues and environments. Fido and Richardson (2019) further demonstrated that empathy mediates the relationship between nature connectedness and prosocial tendencies, confirming that environmental contact enhances empathetic feelings, which in turn stimulate pro-environmental cognition. Di Fabio and Bucci (2016) extended this notion within the green positive psychology framework, proposing that interaction with natural environments elicits emotional wellbeing and ecological awareness, both of which are essential antecedents of empathy. Moreover, Cialdini et al. (1997) proposed the oneness model of empathy, suggesting that perspective-taking and perceived unity with nature reduce self-other boundaries, allowing emotional responses toward nature to mirror altruistic empathy toward people. Collectively, these studies provide a coherent theoretical foundation for arguing that natural environmental perceptions in heritage tourism contexts can positively evoke empathy with nature by stimulating emotional resonance, aesthetic appreciation, and a sense of psychological unity. Thus, we hypothesize:

*H1*: The NE in nature heritage tourism has a positive effect on EN.

#### 2.2.2 Cultural environment and empathy with nature

Beyond the biophysical environment, the cultural environment (CE)—including local traditions, heritage narratives, architecture, rituals, and symbolic meanings—also profoundly influences visitors’ empathetic engagement with nature. As the cultural and natural dimensions of heritage sites are often intertwined, cultural expressions can act as interpretive channels through which nature’s moral and aesthetic values are communicated to visitors. Wu et al. (2020) demonstrated that cultural tourism spaces such as temples create immersive, emotionally charged settings that elicit feelings of reverence, humility, and connection—emotions that overlap conceptually with empathy. Similarly, Zhang S. et al. (2023) argued that linear cultural heritage sites, by integrating historical narratives and spatial symbolism, foster emotional resonance and place attachment among visitors, deepening their empathetic understanding of the human–nature relationship. Zhang and Lee (2022) further highlighted that authenticity perception in cultural heritage contexts enhances visitors’ emotional connection and reduces alienation, enabling them to experience cultural and environmental harmony as part of a shared emotional field.

From a broader socioecological standpoint, the integration of cultural ecosystem services—spiritual fulfillment, aesthetic pleasure, and identity formation—also links cultural perception to empathetic engagement. Ghermandi et al. (2020) demonstrated that heritage landscapes delivering rich cultural ecosystem services evoke affective and symbolic appreciation, revealing that culture serves as an emotional mediator connecting people to their environments. Theoretical contributions from moral psychology echo this notion: Aaltola (2018) emphasized that empathy can arise from ethical reflection and symbolic resonance, not only direct sensory experiences, implying that culturally mediated meanings can cultivate empathetic concern for the natural world. The empathy–altruism model (Cialdini et al., 1997) also supports this process, suggesting that shared identity and symbolic “oneness” foster affective extension beyond the self, whether toward human others or the broader ecological community.

In sum, the cultural environment functions as an interpretive and affective lens through which visitors internalize environmental meanings and experience emotional communion with nature. By evoking spiritual reverence, moral reflection, and symbolic identification, cultural contexts within heritage tourism can significantly enhance empathy with nature. Therefore, it is hypothesized that:

*H2*: The CE in nature heritage tourism can positively stimulate tourists’ EN.

### 2.3 Empathy with nature and tourists’ responsible behaviors

Empathy with nature has been increasingly recognized as a fundamental psychological mechanism driving individuals’ pro-environmental and prosocial behaviors in tourism and environmental psychology research. It reflects the extent to which individuals can emotionally connect with and take the perspective of non-human elements of the natural world (Tam, 2013; Di Fabio and Kenny, 2021). From the empathy-altruism hypothesis (Batson et al., 2015), empathy evokes altruistic motivation that extends beyond interpersonal contexts to include moral concern for the natural environment (Berenguer, 2010). When tourists experience empathy toward nature during their visits, they tend to feel a moral obligation to minimize harm, conserve resources, and contribute positively to the destinations they visit, forming a psychological basis for responsible tourism behaviors.

Empirical evidence has shown that empathy with nature exerts a significant influence on pro-environmental behavioral intentions and actual practices. Schultz (2000) demonstrated that perspective-taking with nature increases individuals’ environmental concern and willingness to engage in protective actions. Similarly, Mayer and Frantz (2004) and Mackay and Schmitt (2019) confirmed that individuals with a higher sense of connectedness to nature exhibit stronger tendencies toward sustainable consumption, waste reduction, and ecological stewardship. Fido and Richardson (2019) further found that empathy mediates the relationship between nature connectedness and moral dispositions such as caring and compassion, suggesting that empathy functions as an internalized moral driver of environmentally responsible actions. Building on this foundation, Wang et al. (2023) empirically verified that empathy with nature significantly enhances tourists’ pro-environmental behavioral intentions, supporting its key role in promoting sustainable behavior in leisure and tourism settings.

In the context of economic responsibility behaviors (ERB)—including behaviors such as supporting local products, paying conservation fees, and contributing financially to community-based environmental initiatives—empathy with nature serves as an intrinsic motivator that transforms emotional resonance with the environment into tangible financial support (Lin et al., 2022; Gao et al., 2017). When tourists empathize with nature, they are more likely to recognize the interdependence between their consumption and the sustainability of the destination, thus internalizing economic responsibility as a form of moral reciprocity (Van Aaken et al., 2013). This mechanism aligns with the empathy-altruism framework, in which empathic concern evokes an altruistic motivation to act in ways that preserve or enhance the welfare of others—including nature as a moral entity (Cialdini et al., 1997). Therefore, empathy with nature can be expected to stimulate tourists’ economic responsibility behaviors.

At the same time, empathy with nature also promotes non-economic responsibility behaviors (NERB)—those behaviors that do not directly involve financial commitment but reflect ethical, environmental, and social responsibility, such as obeying regulations, avoiding environmental damage, and spreading environmental awareness (Gao et al., 2017; Lin et al., 2022). When tourists feel emotionally connected to and compassionate toward the natural world, they tend to experience moral emotions such as guilt or gratitude that guide them toward respectful and protective behaviors (Berenguer, 2007; Tam et al., 2013). Chen et al. (2024) demonstrated that empathy with nature significantly mediates the relationship between place attachment and pro-environmental behavior, emphasizing its role in translating affective connection into concrete actions. Similarly, Fritsche et al. (2018) in their Social Identity Model of Pro-Environmental Action (SIMPEA) proposed that emotional identification with nature leads to a collective sense of environmental responsibility, which extends to non-economic behavioral intentions such as following ecological norms and encouraging others to act sustainably.

Thus, empathy with nature provides both affective and moral foundations for responsible behaviors in tourism contexts. It activates moral emotions and prosocial orientations that encourage tourists to engage in both economic and non-economic actions that benefit the environment and local communities. Accordingly, this study proposes that empathy with nature helps stimulate tourists’ economic responsibility behaviors (H3) and non-economic responsibility behaviors (H4).

*H3*: Empathy with nature helps stimulate tourists’ economic responsibility behaviors.

*H4*: Empathy with nature helps stimulate tourists’ non-economic responsibility behaviors.

### 2.4 Mediating roles of empathy with nature

In natural heritage tourism contexts, the influence of environmental perceptions—both natural and cultural—on tourists’ responsible behavioral intentions is not always direct. A growing body of research suggests that empathy with nature functions as a crucial psychological mechanism mediating this relationship (e.g., Fido and Richardson, 2019; Zhang Z. et al., 2023). This mediating effect reflects the process by which environmental experiences are internalized, transformed into emotional and moral responses, and subsequently guide responsible actions. Specifically, when tourists perceive high-quality natural or culturally rich environments, these perceptions can evoke empathic engagement with nature, which in turn translates into both economic responsibility behaviors (ERB) and non-economic responsibility behaviors (NERB).

#### 2.4.1 Natural environment → Empathy with nature → Responsible behaviors

Empirical evidence supports the mediating role of empathy with nature between natural environmental perceptions and responsible behaviors. Fido and Richardson (2019) demonstrated that empathy acts as a psychological conduit between nature connectedness and prosocial dispositions, suggesting that emotional engagement is essential for translating environmental perception into behavioral intention. Wang et al. (2023) further confirmed that empathy with nature mediates the impact of natural exposure on tourists’ pro-environmental behaviors, highlighting that sensory and aesthetic experiences of the natural environment first elicit affective resonance, which then motivates ethical and protective actions. In the context of economic responsibility behaviors, empathy encourages tourists to financially support conservation efforts or local sustainability initiatives as a moral response to the perceived needs of the environment (Lin et al., 2022). Similarly, for non-economic responsibility behaviors, empathic engagement with natural settings fosters normative adherence, ecological mindfulness, and ethical conduct toward the environment (Chen et al., 2024; Berenguer, 2007).

#### 2.4.2 Cultural environment → empathy with nature → responsible behaviors

The mediating mechanism is equally applicable to cultural environmental perceptions. Cultural environments, encompassing historical sites, intangible heritage, and locally embedded narratives, provide symbolic and moral cues that evoke empathic responses toward nature. Wu et al. (2020) highlighted that interactive cultural experiences in tourism spaces can cultivate moral and emotional resonance with both culture and environment. Zhang Z. et al. (2023) and Zhang and Lee (2022) similarly emphasized that authenticity and interpretive depth in cultural heritage enhance tourists’ emotional engagement, fostering empathy that extends to environmental stewardship. Through this pathway, cultural environment perceptions indirectly shape both ERB and NERB: tourists who emotionally resonate with the moral and symbolic significance of cultural heritage are more likely to adopt responsible behaviors, whether through supporting sustainable tourism economically or by adhering to ethical and conservation norms (Ghermandi et al., 2020).

#### 2.4.3 Theoretical integration

The mediating role of empathy with nature aligns with the empathy-altruism hypothesis (Batson et al., 2015), which posits that empathic concern generates motivation for prosocial actions. In tourism contexts, this process translates environmental perceptions into behavioral intentions, with empathy serving as the affective and moral conduit. The Social Identity Model of Pro-Environmental Action (SIMPEA) (Fritsche et al., 2018) further explains that empathic identification with nature fosters a collective sense of environmental responsibility, bridging individual perception with both economic and non-economic actions. Collectively, these theoretical and empirical insights provide strong justification for the proposed mediation hypotheses:

*H5a*: EN plays a mediate role between NE and ERB.

*H5b*: EN plays a mediate role between NE and NERB.

*H5c:* EN plays a mediate role between CE and ERB.

*H5d*: EN plays a mediate role between CE and NERB.

### 2.5 Moderating roles of perceived escapism

Perceived escapism (PE) represents a psychological state in which individuals temporarily detach from their everyday realities to seek immersion, fantasy, or self-projection (Hirschman, 1983). In tourism contexts, escapism is a key motivator influencing how visitors perceive and emotionally respond to natural and cultural environments. By allowing tourists to immerse themselves fully in the tourism experience, a sense of escapism can enhance their attentional focus, emotional receptivity, and cognitive openness, thereby potentially amplifying affective responses such as empathy with nature (Orazi et al., 2023). Escapism thus serves not merely as a passive diversion but as an active psychological mechanism that can shape the intensity of emotional engagement with environmental stimuli.

Empirical and theoretical studies support the moderating role of escapism in leisure and consumer experiences. Hirschman (1983) proposed that escapism enables individuals to project themselves into alternative realities, intensifying emotional and cognitive engagement with the stimuli encountered. In tourism research, Kim et al. (2020) found that immersive experiences in virtual reality tourism heightened both affective responses and behavioral intentions through the mechanism of escapism. Although their study focused on digital tourism, the underlying principle is transferable: tourists experiencing a high sense of escapism in natural or cultural heritage settings are likely to engage more deeply with the environment, thereby enhancing empathy with nature. Similarly, Mirowska and Arsenyan (2023) demonstrated that escapism amplifies the emotional resonance of empathic experiences, suggesting that the psychological detachment from everyday life fosters greater affective responsiveness. Even studies in immersive rehabilitation contexts highlight that engaging, escapist experiences can significantly increase emotional and attentional engagement (Huang et al., 2024), underscoring the potential of escapism to modulate experiential outcomes.

Within natural heritage tourism, escapism is expected to interact with both natural and cultural environmental perceptions. For the natural environment, tourists who experience higher levels of escapism are more likely to be fully absorbed in the sensory and aesthetic richness of landscapes, wildlife, and ecological features, which in turn strengthens their empathic responses to the environment (Hirschman, 1983; Orazi et al., 2023). Regarding the cultural environment, escapism allows tourists to mentally immerse themselves in historical narratives, rituals, and symbolic meanings, intensifying emotional resonance and moral identification with the human–nature connection embedded in heritage sites (Kim et al., 2020; Mirowska and Arsenyan, 2023). In both cases, perceived escapism amplifies the affective processing of environmental cues, suggesting that the relationship between environmental perceptions and empathy with nature is contingent upon the degree of escapism experienced by tourists.

Consequently, perceived escapism is posited as a moderator that strengthens the impact of both natural and cultural environmental perceptions on empathy with nature. Specifically, tourists who experience high levels of escapism are expected to exhibit more intense empathic engagement with nature in response to environmental stimuli compared to those with low escapism. This provides a theoretical foundation for the following hypotheses:

*H6a*: PE plays a moderating role between NE and EN.

*H6b*: PE plays a moderating role between CE and EN.

### 2.6 Moderating roles of awe

Awe is a complex emotional response characterized by a sense of vastness and a need for cognitive accommodation, often elicited by experiences in nature, art, or significant cultural phenomena (Keltner and Haidt, 2003). In tourism contexts, awe emerges when visitors encounter landscapes, heritage sites, or wildlife that surpass their ordinary frames of reference, evoking profound emotional and cognitive engagement (Piff et al., 2015). Importantly, awe has been shown to enhance prosocial motivations and ethical sensitivity, thereby influencing environmentally responsible behaviors (Wang and Lu, 2024).

Empirical and theoretical research highlights that awe can amplify the impact of empathy on behavioral intentions. Piff et al. (2015) demonstrated that awe induces the “small self” phenomenon, reducing self-centeredness and increasing concern for others, which parallels tourists’ enhanced consideration for natural and cultural environments. Similarly, Prade and Saroglou (2016) found that awe promotes generosity and helping behaviors, suggesting that emotionally elevated states translate into both economic and non-economic prosocial actions. Within tourism settings, Wang and Lu (2024) specifically examined wildlife tourists, showing that awe enhances empathy with nature, which in turn fosters animal-friendly behavioral intentions, providing direct empirical evidence for awe as a mechanism that strengthens the empathy–behavior relationship.

The moderating role of awe is particularly relevant when considering the link between empathy with nature and tourists’ responsible behaviors. For economic responsibility behaviors (ERB), such as supporting conservation fees, local eco-products, or sustainable tourism services, tourists who experience high levels of awe are more likely to translate empathic feelings into concrete financial support for environmental sustainability (Qi et al., 2018). Regarding non-economic responsibility behaviors (NERB), including normative adherence, ecological mindfulness, and advocacy for environmental protection, awe intensifies the moral salience of empathic engagement with nature, leading to higher likelihood of ethical and protective actions (Yam et al., 2023). By heightening both moral and emotional sensitivity, awe reinforces the motivational power of empathy, shaping tourists’ behavioral outcomes in both economic and non-economic dimensions.

Consequently, awe is proposed to act as a moderator that strengthens the effect of empathy with nature on both types of responsible behaviors. Tourists experiencing high levels of awe are expected to exhibit stronger translation of empathic engagement into concrete behavioral intentions compared to those experiencing low levels of awe. This provides a theoretical basis for the following hypotheses:

*H7a*: Awe plays a moderate role between tourists’ EN and ERB.

*H7b*: Awe plays a moderate role between tourists’ EN and NERB.

### 2.7 Proposed research model

**FIGURE 1:**
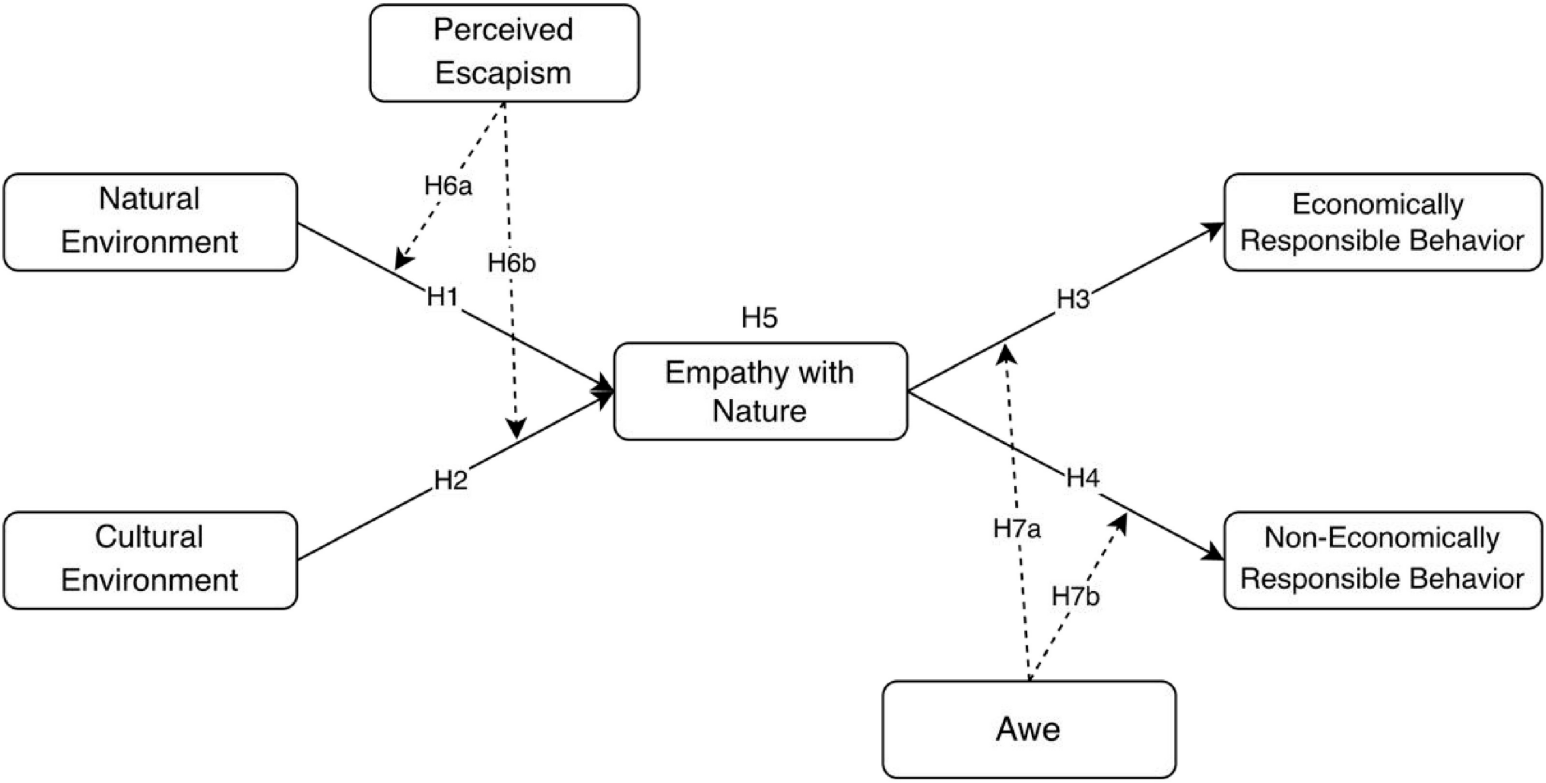
Research model.

The research model of this study is shown in Figure 1.

## 3 Materials and methods

### 3.1 Study site

This study focuses on the Shennongjia Scenic Area as its research object. Located in the western part of Hubei Province, China, Shennongjia is a tourist destination that integrates both natural landscapes and cultural heritage. Its rich ecological resources and unique natural scenery have made it a popular destination for many tourists. From the perspective of nature heritage tourism, Shennongjia not only boasts abundant natural landscapes but also has a deep cultural foundation.

### 3.2 Questionnaire design

The questionnaire in this study was designed to comprehensively capture participants’ demographic characteristics, experiences in the Shennongjia Scenic Area, and psychological and behavioral responses after their visit. It consists of two main sections. The first section collects basic demographic information, including participants’ age, gender, education level and salary. The second section focuses on tourists’ experiences and psychological responses during and after their visit, specifically measuring perceptions of the natural and cultural environment (e.g., The natural environment of Shennongjia makes me feel the grandeur of nature; The folk legends of Shennongjia, such as the story of Shennong tasting hundreds of herbs, deeply move me), empathy with nature (e.g., Being in the Shennongjia forest area makes me feel a strong bond with the natural environment; Being in the Shennongjia forest area fosters an internal sense of connection between humans and nature), perceived escapism (e.g., Visiting Shennongjia makes me feel as if I am in another world), awe (e.g., The Shennongjia forest area evokes a feeling of humility), and post-visit responsible behavioral intentions (e.g., I am willing to purchase souvenirs to contribute economically to Shennongjia; I am willing to protect the scarce natural and cultural resources of Shennongjia).

These survey questions were initially developed based on established scales (e.g., Tam, 2013) or directly relevant literature (e.g., Orazi et al., 2023; Piff et al., 2015) in English, professionally translated into clear and accurate Chinese, and finally administered and collected in Chinese. Each construct was assessed using multiple items measured on a seven-point Likert scale ranging from 1 (“strongly disagree”) to 7 (“strongly agree”), in line with established practices in environmental psychology and tourism research.

### 3.3 Data collection

Prior to the formal survey, a pilot study with 56 valid responses was conducted to evaluate the reliability and validity of the scales, confirming their suitability for the main study. The formal survey was distributed through two channels: offline distribution by research team members to acquaintances who had visited Shennongjia, and online distribution via the Wenjuanxing platform^1^ targeting the general public with prior visitation experience. In total, 653 questionnaires were collected, of which 525 were deemed valid after excluding incomplete or inattentive responses, resulting in a valid response rate of 80.4%. The collected data were subsequently analyzed to ensure the reliability and validity of the constructs and to prepare for further statistical modeling, including confirmatory factor analysis and structural equation modeling.

## 4 Results

### 4.1 Sample profile

Summary of participants’ basic information (see Table 1).

**TABLE 1:** Demographics profile of the sample (*n* = 525).

Variable	Category	Frequency	%
Gender	Male	262	49.9
	Female	263	50.1
Age	18–25	183	34.9
	26–35	141	26.9
	36–45	107	20.4
	46–55	66	12.6
	>55	28	5.3
Education	High school and below	64	12.2
	College/junior college	312	59.4
	Post-graduate	107	20.4
	Doctor and beyond	42	8.0
Salary (Yuan)	<5,000	231	44.0
	5,000–8,000	160	30.5
	8,001–10,000	49	9.3
	10,001–15,000	56	10.7
	>15,000	29	5.5

A notable feature of the present study’s sample is the relatively high educational level of respondents, with nearly 90% possessing a university degree or higher, as well as the relatively young age profile, with over 61% under 35 years old and more than 82% under 45. These characteristics may raise questions regarding the representativeness of the sample for the broader population of natural heritage tourists.

Several factors help contextualize and interpret these patterns. First, prior research on natural heritage tourism indicates that visitors to ecologically and culturally significant sites often skew toward younger and more highly educated demographics. Young adults and middle-aged individuals tend to have greater leisure mobility, higher interest in cultural and ecological experiences, and greater access to information through digital platforms, making them more likely to participate in heritage tourism activities (Zhang Z. et al., 2023; Tian et al., 2023). Similarly, higher education levels are associated with greater environmental awareness, cultural interest, and willingness to engage in responsible behaviors, which aligns with the profile observed in the current sample (Berenguer, 2007; Mackay and Schmitt, 2019).

Second, the age and education distribution may partially reflect the specific characteristics of the Shennongjia destination and the study’s survey methodology. Shennongjia, as a nature–cultural heritage site with relatively remote access and structured tourism services, naturally attracts visitors with higher socioeconomic resources, travel literacy, and information-seeking motivation. Moreover, the use of structured questionnaires and on-site sampling can inadvertently favor visitors who are literate, comfortable with survey participation, and willing to allocate time for structured response, further contributing to the observed demographic profile.

Third, while the sample is skewed toward younger and highly educated visitors, this does not necessarily invalidate the study’s findings. The primary objective of this research is to examine the psychological mechanisms linking environmental perceptions, empathy with nature, and responsible behavioral intentions. Prior literature indicates that these mechanisms are generally robust across adult populations, although effect magnitudes may vary with age or education (Di Fabio and Kenny, 2021; Wang et al., 2023). Nevertheless, the findings may be most directly applicable to the demographic segments most likely to visit and actively engage with natural heritage tourism sites.

### 4.2 Confirmatory factor analysis

The convergent validity of the scales is in Table 2.

**TABLE 2:** Convergent validity.

Variable	Items	Factor loading	Cronbach’s α	CR	AVE
Natural environment	NE1	0.827	0.887	0.888	0.664
	NE2	0.790			
	NE3	0.793			
	NE4	0.848			
Cultural environment	CE1	0.801	0.879	0.879	0.646
	CE2	0.777			
	CE3	0.797			
	CE4	0.839			
Empathy with nature	EN1	0.854	0.936	0.936	0.746
	EN2	0.877			
	EN3	0.865			
	EN4	0.864			
	EN5	0.857			
Perceived escapism	PE1	0.835	0.895	0.895	0.681
	PE2	0.828			
	PE3	0.819			
	PE4	0.819			
Awe	AWE1	0.875	0.933	0.933	0.737
	AWE2	0.881			
	AWE3	0.817			
	AWE4	0.868			
	AWE5	0.849			
Economically responsible behavior	ERB1	0.825	0.891	0.892	0.673
	ERB2	0.797			
	ERB3	0.836			
	ERB4	0.823			
Non-economically responsible behavior	NERB1	0.867	0.943	0.942	0.766
	NERB2	0.862			
	NERB3	0.874			
	NERB4	0.878			
	NERB5	0.895			

Table 2 shows that the overall scale has a relatively ideal convergent validity (Hair et al., 1998). Based on this, a further examination of discriminant validity shows in Table 3.

**TABLE 3:** Discriminant validity.

Items	Mean.	S.D.	NE	CE	EN	PE	AWE	ERB	NERB
NE	5.453	1.270	**0.815**						
CE	5.274	1.198	0.522	**0.804**					
EN	5.399	1.261	0.537	0.529	**0.864**				
PE	5.227	1.306	0.333	0.272	0.415	**0.825**			
AWE	5.303	1.266	0.262	0.324	0.344	0.292	**0.858**		
ERB	5.173	1.324	0.522	0.544	0.512	0.327	0.283	**0.820**	
NERB	5.540	1.275	0.415	0.472	0.501	0.275	0.269	0.396	**0.875**

The bold values on the diagonal represent the square roots of the AVE for each latent variable, while the values below the diagonal denote the correlations among the latent variables. It presents the discriminant validity results, showing that all inter-construct correlations are lower than the corresponding square roots of the AVE on the diagonal, indicating satisfactory discriminant validity among the variables.

From Table 3 we can see that discriminant validity among the variables meets the requirements (Fornell and Larcker, 1981).

### 4.3 Structural equation modeling

**FIGURE 2:**
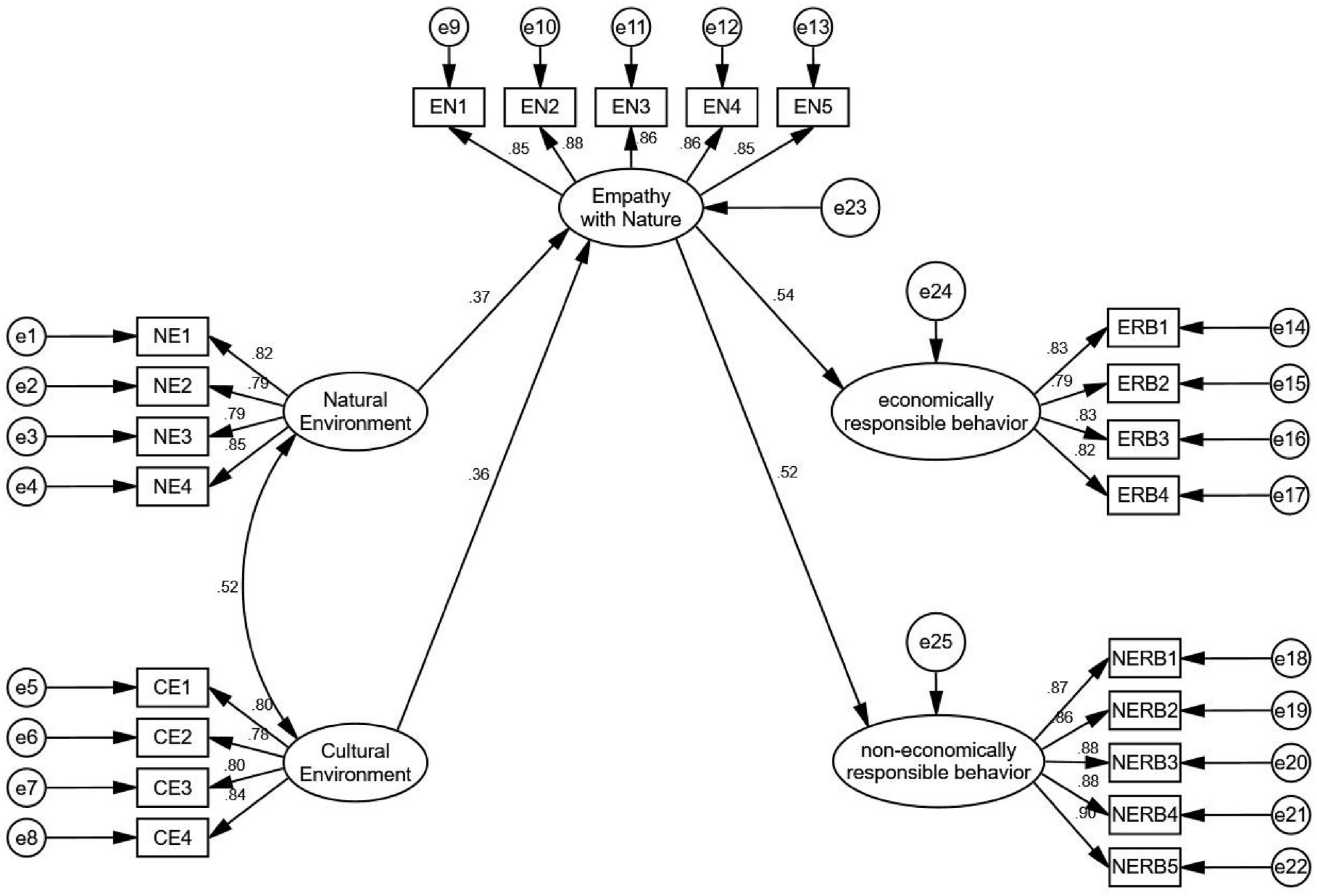
Results of SEM.

Analysis above confirmed that the reliability and validity all meet the requirements. Then we utilize AMOS 24.0 to construct SEM diagram (see Figure 2 and Table 4).

**TABLE 4:** Model fit.

Classification	X^2^/df	RMSEA	IFI	TLI	CFI	PGFI	PNFI
Criteria	<3	<0.08	>0.9	>0.9	>0.9	>0.5	>0.5
Value	1.978	0.043	0.977	0.974	0.977	0.753	0.843

Table 4 shows that in terms of absolute fit indices (χ^2^/df, RMSEA), incremental fit indices (IFI, TLI, CFI), and parsimonious fit indices (PGFI, PNFI), all indicators reached acceptable levels, indicating that the overall model fit meets the required standards. Based on this, further path analysis was conducted (see Table 5).

**TABLE 5:** SEM results.

H	Path			β	S.E.	C.R.	Cohen’s f^2^	*P*	Supported
H1	EN	< —	NE	0.367	0.048	7.419	0.135	***	Yes
H2	EN	< —	CE	0.358	0.052	7.195	0.121	***	Yes
H3	ERB	< —	EN	0.536	0.049	11.583	0.288	***	Yes
H4	NERB	< —	EN	0.519	0.046	11.684	0.285	***	Yes

***p < 0.001.

Table 5 shows that all the hypotheses are supported. On this basis, further tests will be conducted on the mediating and moderating effects.

### 4.4 Meditation effect

We employed the bootstrapping method to do the mediating effects test seeing the result below in Table 6.

**TABLE 6:** Mediation effect.

	95%CI				
Path	Estimate	SE	Lower	Upper	Result
NE→EN→ERB	0.197	0.045	0.118	0.290	Support
NE→EN→NERB	0.190	0.041	0.117	0.279	Support
CE→EN→ERB	0.192	0.042	0.110	0.278	Support
CE→EN→NERB	0.186	0.045	0.106	0.285	Support

Table 6 indicates that the mediating paths through NE → EN → ERN/NERB are all significant: [estimate = 0.197, SE = 0.045, 95% CI: (0.118, 0.290); estimate = 0.190, SE = 0.041, 95% CI: (0.117, 0.279)], thereby validating H5a and H5b.

Similarly, the mediating paths through CE → EN → ERN/NERB are all significant: [estimate = 0.192, SE = 0.042, 95% CI: (0.110, 0.278); estimate = 0.186, SE = 0.045, 95% CI: (0.106, 0.285)], H5c and H5d are also proved.

### 4.5 Moderating effect

**FIGURE 3:**
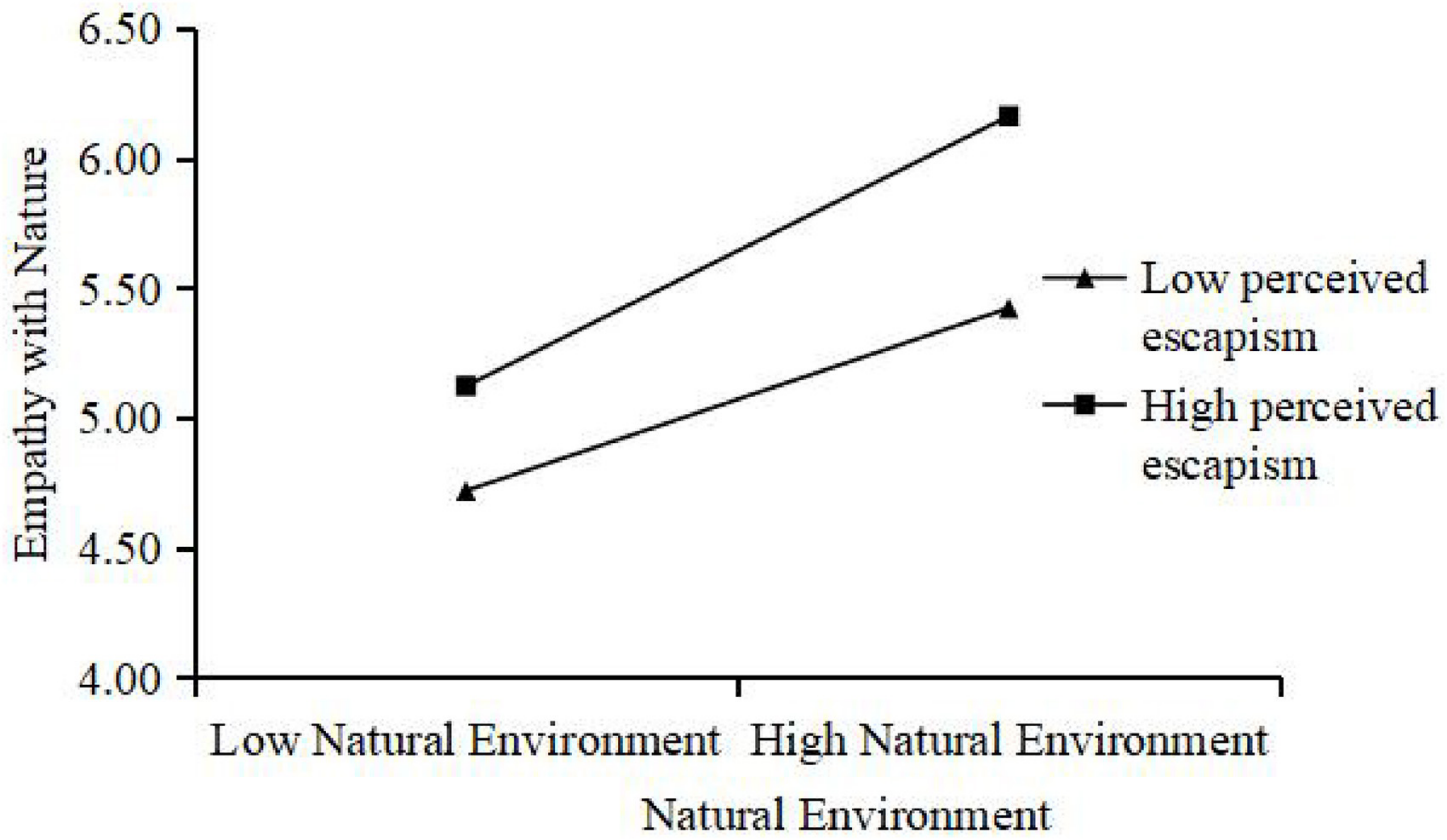
Moderating role of PE with NE and EN.

Last we tested the moderating effect of the two factors. First, we examined the moderating role of PE between both NE and CE and EN. The results tell that PE positively moderates the relationship between NE and EN (β = 0.145, SE = 0.023, *P* < 0.001). The interaction effects are illustrated in Figure 3, which validate Hypotheses H6a. However, no significant interaction was found between CE and EN (β = –0.038, SE = 0.024, *P* > 0.05), thus Hypothesis H6b is not supported.

**FIGURE 4:**
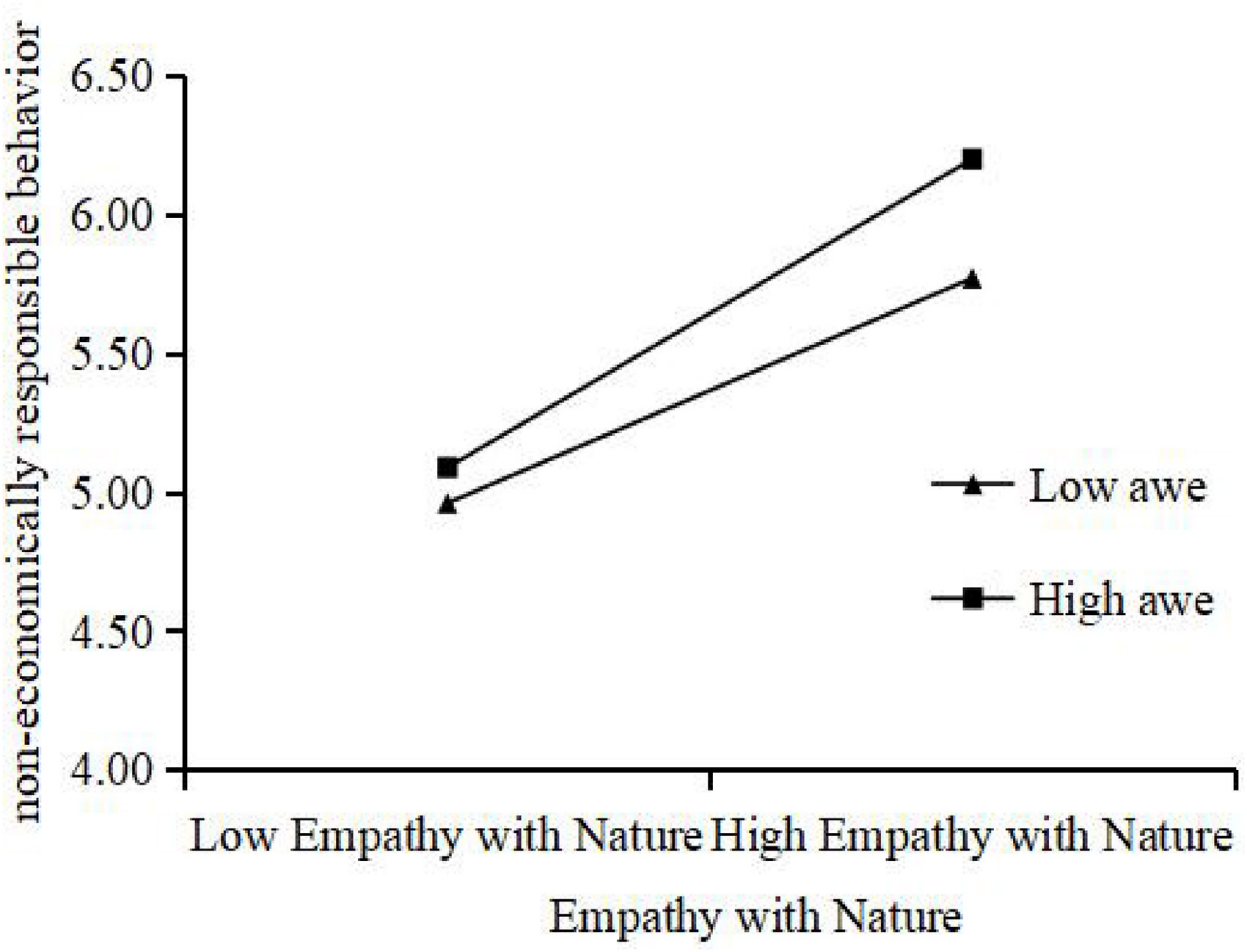
Moderating role of AWE with EN and NERB.

We also tested the moderating role of AWE between EN and both ERB and NERB. The results reveal a positive interaction between AWE and EN on NERB (β = 0.135, SE = 0.023, *P* < 0.001), as it showed in Figure 4, which validate Hypotheses H7b. However, no significant interaction was shown between AWE and EN on ERB (β = –0.013, SE = 0.024, *P* > 0.05), thus Hypothesis H7a is not supported.

## 5 Conclusion, discussion, and implications

### 5.1 Conclusion and discussion

The results of this study provide comprehensive insights into the mechanisms through which natural and cultural environmental perceptions influence tourists’ responsible behavioral intentions in natural heritage tourism, with empathy with nature serving as a central mediator and perceived escapism and awe acting as moderators. Overall, the findings support the critical role of affective and cognitive engagement in translating environmental perceptions into responsible behaviors, while highlighting boundary conditions that may influence these effects.

#### 5.1.1 Natural and cultural environment perceptions and empathy with nature (H1–H2)

Both H1 and H2 were supported, indicating that tourists’ perceptions of the natural and cultural environments positively stimulate empathy with nature. Specifically, the natural environment’s aesthetic qualities, biodiversity, and ecological richness were found to directly enhance tourists’ emotional resonance with nature, confirming prior research emphasizing the affective and cognitive pathways of environmental engagement (Schultz, 2000; Tam, 2013; Di Fabio and Kenny, 2021). Similarly, cultural environment perceptions—including historical heritage, local narratives, and authenticity of cultural expressions—were found to significantly promote empathic engagement, in line with studies highlighting the moral and emotional value embedded in cultural heritage (Wu et al., 2020; Zhang Z. et al., 2023). These results underscore the dual importance of natural and cultural stimuli in cultivating emotional bonds with the environment, suggesting that both ecological and cultural elements are integral to fostering empathy-driven responsible behaviors in natural heritage tourism contexts.

#### 5.1.2 Empathy with nature and responsible behavioral intentions (H3–H4)

H3 and H4 were also supported, confirming that empathy with nature positively affects both economic responsibility behaviors (ERB) and non-economic responsibility behaviors (NERB). Tourists who emotionally resonate with the environment are more willing to engage in supportive actions, such as contributing financially to conservation initiatives (ERB) and adhering to ecological norms or engaging in pro-environmental advocacy (NERB), consistent with previous findings (Berenguer, 2007; Wang et al., 2023; Chen et al., 2024). These results reinforce the centrality of empathy as a motivational mechanism linking environmental perceptions to responsible actions, highlighting that affective engagement is critical in shaping both material and normative behaviors toward the environment.

#### 5.1.3 Mediating role of empathy with nature (H5a–H5d)

All four mediation hypotheses (H5a–H5d) were supported, demonstrating that empathy with nature mediates the effects of both natural and cultural environmental perceptions on ERB and NERB. This finding aligns with theoretical propositions from the empathy-altruism hypothesis (Batson et al., 2015) and the Social Identity Model of Pro-Environmental Action (Fritsche et al., 2018), which suggest that emotional resonance with environmental stimuli transforms perceptual inputs into prosocial behavioral intentions. Specifically, the results indicate that tourists do not respond solely to the environmental cues themselves but engage in a psychologically mediated process in which empathy translates perceived environmental value into concrete responsible actions. This highlights the importance for tourism managers of designing experiences that evoke both aesthetic appreciation and emotional engagement to maximize responsible behaviors.

#### 5.1.4 Moderating role of perceived escapism (H6a–H6b)

The results show that H6a was supported, whereas H6b was not. Specifically, perceived escapism strengthened the relationship between natural environment perceptions and empathy with nature, suggesting that tourists who experience a strong sense of escapism are more likely to immerse themselves in natural environments and develop empathic connections, consistent with prior research on immersive experiences and attentional focus (Hirschman, 1983; Orazi et al., 2023). However, perceived escapism did not significantly moderate the effect of cultural environment perceptions on empathy with nature (H6b). A possible explanation is that cultural heritage experiences, unlike natural landscapes, often require cognitive processing of historical knowledge, social narratives, and symbolic meanings. As a result, the affective boost provided by escapism may be less relevant for processing cultural cues, which depend more on interpretive engagement than on immersive detachment from reality. This distinction suggests that the role of escapism as a moderator may be context-dependent, being more effective in facilitating affective engagement with inherently immersive stimuli such as natural environments than with cognitively demanding cultural experiences.

#### 5.1.5 Moderating role of awe (H7a–H7b)

Regarding the moderating effect of awe, H7b was supported while H7a was not. Specifically, awe strengthened the relationship between empathy with nature and non-economic responsibility behaviors, such as normative adherence and ecological mindfulness, consistent with prior findings that awe enhances moral sensitivity and prosocial behaviors (Piff et al., 2015; Qi et al., 2018; Wang and Lu, 2024). However, awe did not significantly moderate the relationship between empathy and economic responsibility behaviors (H7a). One potential explanation is that awe primarily triggers intrinsic motivation and moral elevation, which more directly translates into normative, non-material actions rather than financial support or market-based contributions. Economic behaviors may be influenced more strongly by external factors, such as perceived efficacy, social norms, or financial capacity, which are less susceptible to emotional elevation induced by awe. This finding highlights the nuanced role of awe, suggesting that it selectively enhances morally or socially oriented behaviors rather than material or transactional ones.

In summary, the results provide strong empirical support for the theoretical framework positing empathy with nature as a central mediator between environmental perceptions and responsible behaviors, while identifying boundary conditions in which the moderating effects of escapism and awe are contingent on environmental type and behavioral domain. Natural and cultural perceptions stimulate empathy, which drives both ERB and NERB, and perceived escapism enhances the empathy response to natural environments. Awe, in contrast, selectively strengthens empathy’s impact on non-economic, morally oriented behaviors, revealing important nuances in the emotional drivers of sustainable tourism behavior.

### 5.2 Theoretical implications

This study contributes to the literature on natural heritage tourism, environmental psychology, and responsible tourism behavior in several important ways. First, it extends the theoretical understanding of the mechanisms linking environmental perceptions to tourists’ responsible behavioral intentions by empirically demonstrating the central mediating role of empathy with nature. While previous studies have emphasized the importance of connectedness to nature in shaping pro-environmental behaviors (Schultz, 2000; Mayer and Frantz, 2004; Tam, 2013), this research provides nuanced evidence that empathy functions as a psychological conduit that translates both natural and cultural environmental stimuli into concrete behavioral outcomes. By showing that empathy mediates the effects of both environmental types on economic and non-economic responsibility behaviors, the study reinforces the applicability of the empathy-altruism hypothesis (Batson et al., 2015) and the Social Identity Model of Pro-Environmental Action (SIMPEA) (Fritsche et al., 2018) in tourism contexts, bridging a gap between theoretical constructs and real-world tourism experiences.

Second, this research advances the understanding of dual environmental stimuli—natural and cultural environments—in shaping emotional and behavioral responses. Previous research often focused on either natural landscapes or cultural heritage independently (Zhang Z. et al., 2023; Wu et al., 2020), but the present study demonstrates that both environmental types contribute to fostering empathy with nature, which in turn motivates responsible behaviors. This integrative perspective highlights that sustainable tourism cannot rely solely on ecological or cultural interventions; rather, the combined stimulation of natural and cultural cues is critical for eliciting affective engagement and promoting both economic and normative responsibility behaviors among tourists.

Third, the study provides new insights into the boundary conditions under which environmental perceptions influence empathy. By testing the moderating effects of perceived escapism and awe, the findings reveal important contingencies in these emotional processes. Specifically, perceived escapism was found to amplify the influence of natural environment perceptions on empathy but not that of cultural environment perceptions. This distinction underscores the role of immersive, affectively rich stimuli in facilitating empathy and suggests that cognitive engagement with cultural heritage may be less sensitive to escapist detachment. Similarly, awe selectively strengthened the relationship between empathy and non-economic responsibility behaviors but not economic responsibility behaviors. This finding elucidates the differential effects of moral-elevating emotions, suggesting that awe primarily enhances ethically and socially motivated behaviors rather than financial or transactional actions. These results extend previous work on awe in tourism and environmental psychology (Piff et al., 2015; Qi et al., 2018; Wang and Lu, 2024), highlighting its selective efficacy in influencing morally oriented behavioral outcomes.

Finally, the findings contribute to theory by differentiating the motivational pathways of economic and non-economic responsibility behaviors in natural heritage tourism. While prior studies often treated pro-environmental behavior as a monolithic construct, this research demonstrates that affective mechanisms, such as empathy and awe, interact with contextual factors to influence different behavioral domains in distinct ways. Economic behaviors, being more instrumental and potentially constrained by resources, are less sensitive to awe-induced moral elevation, whereas non-economic behaviors, which are normatively and morally grounded, are more strongly influenced by affective states. This distinction offers a more granular theoretical understanding of the emotional and cognitive antecedents of responsible tourism behavior.

In sum, the present study provides a comprehensive theoretical framework linking environmental perceptions, empathy with nature, and tourists’ responsible behaviors, while identifying the nuanced moderating roles of escapism and awe. It bridges gaps between environmental psychology, cultural tourism, and sustainable behavior research, offering an empirically grounded and contextually relevant contribution to the theoretical literature on natural heritage tourism and pro-environmental behavior.

### 5.3 Practical implications

The findings of this study provide several actionable insights for tourism practitioners, heritage site managers, and policymakers aiming to promote responsible tourism behaviors in natural heritage destinations.

#### 5.3.1 Enhancing natural and cultural environmental experiences

Given that both natural and cultural environmental perceptions significantly stimulate empathy with nature (H1–H2), tourism managers should focus on enhancing the quality and accessibility of environmental stimuli. For natural environments, this may include preserving biodiversity, maintaining scenic integrity, and designing immersive interpretive trails that allow visitors to experience ecological richness first-hand. For cultural environments, providing authentic, well-curated exhibits, interactive storytelling, and opportunities for participatory cultural engagement can strengthen tourists’ emotional connection to heritage. The dual emphasis on natural and cultural elements ensures that tourists are cognitively and affectively engaged, thereby promoting both economic and non-economic responsibility behaviors (H3–H4; H5a–H5d).

#### 5.3.2 Designing experiences that foster empathy

Since empathy with nature mediates the relationship between environmental perceptions and responsible behaviors, tourism experiences should be deliberately designed to evoke emotional engagement. Interpretive signage, guided tours, and interactive educational programs can be employed to highlight ecological and cultural significance, encouraging visitors to reflect on their emotional and ethical responsibilities. Storytelling techniques that link environmental and cultural narratives with personal relevance can further enhance empathic engagement, translating affective responses into concrete behaviors such as conservation support or adherence to environmental norms.

#### 5.3.3 Leveraging perceived escapism for natural environment engagement

The study found that perceived escapism amplifies the effect of natural environment perceptions on empathy (H6a), suggesting that immersive and psychologically engaging experiences can enhance affective responses. Destination managers can design spaces and experiences that facilitate escapism, such as secluded nature trails, scenic viewpoints, or virtual reality-enhanced interpretive experiences. These approaches allow visitors to temporarily detach from routine life, immerse themselves in the natural environment, and develop stronger empathic connections. However, as escapism did not significantly moderate the impact of cultural environments (H6b), strategies aimed at cultural engagement may need to focus more on interpretive depth and cognitive stimulation rather than immersive escapism.

#### 5.3.4 Cultivating awe to promote non-economic responsibility behaviors

The results indicate that awe strengthens the relationship between empathy and non-economic responsibility behaviors (H7b), highlighting its utility in encouraging normatively driven, ethical actions. Managers can create conditions for awe by showcasing monumental landscapes, rare wildlife encounters, or culturally significant rituals and performances. Interactive interpretation that emphasizes the scale, history, or uniqueness of heritage can also evoke awe. While awe may be less effective for economic responsibility behaviors (H7a), its role in promoting ecological mindfulness, conservation advocacy, and normative compliance underscores its importance for sustainable behavior promotion.

#### 5.3.5 Differentiated strategies for economic and non-economic behaviors

The differential effects of empathy and awe on economic versus non-economic behaviors suggest that tailored strategies are needed. To promote economic responsibility behaviors, destinations might combine empathy-inducing experiences with practical incentives, such as donation programs, eco-certification schemes, or opportunities to support local sustainable products. For non-economic behaviors, emotional and moral experiences, particularly those that evoke awe or moral elevation, should be emphasized, as these are more likely to translate affective engagement into voluntary ethical actions.

#### 5.3.6 Integrated design for sustainable heritage tourism

Overall, these findings support an integrated design approach that simultaneously addresses natural and cultural stimuli, empathy induction, and affective amplification through escapism and awe. By aligning experiential design with psychological mechanisms, heritage destinations can more effectively foster both economic and non-economic responsible behaviors, contributing to long-term sustainability and visitor satisfaction.

## 6 Limitations and future research directions

Despite the significant theoretical and practical contributions of this study, several limitations should be acknowledged, which also provide directions for future research.

### 6.1 Sample representativeness

The study sample consisted predominantly of younger and highly educated visitors, with nearly 90% holding a university degree or higher and over 61% under 35 years old. Although this demographic profile aligns with typical visitors to Shennongjia and similar natural heritage destinations, it may limit the generalizability of the findings to older or less formally educated tourist segments. Future studies could adopt stratified sampling or multi-site survey strategies to capture a broader range of visitor characteristics and test whether the proposed psychological mechanisms remain robust across diverse demographic groups.

### 6.2 Contextual influences of natural heritage tourism

Natural heritage tourism involves multiple experiential components beyond the natural environment itself—such as perceived prestige of UNESCO status, educational framing, conservation messaging, cultural narratives, and varying levels of engagement during the visit. These contextual factors were not controlled in this study but may shape visitors’ emotional responses, cognitive evaluations, and even their empathy with nature. Consequently, the observed pathways (e.g., environmental perceptions → empathy with nature → responsible behavior) should be interpreted with caution, as they may be partially influenced by these co-occurring factors. Future research could incorporate measures of perceived prestige, educational involvement, or interpretive engagement to more precisely disentangle their effects and assess the stability of the proposed model across different interpretive or managerial conditions.

### 6.3 Cross-sectional design

Because the study relies on cross-sectional self-report data, definitive causal claims cannot be established. While structural equation modeling offers a rigorous test of the hypothesized associations and mediation effects, longitudinal, pre–post visit, or experimental designs are needed to further validate the causal direction of the mediating role of empathy with nature. Future studies may manipulate environmental cues, interpretive content, or emotional stimuli to better assess causal pathways.

### 6.4 Measurement limitations

Although validated scales were used to assess empathy with nature, responsible behavioral intentions, perceived escapism, and awe, self-reported measures are susceptible to social desirability bias and may not fully capture actual behavior. Future studies could integrate behavioral observation, digital behavioral tracking, or experimental simulations to complement self-reported data and enhance the ecological validity of the findings.

Addressing these limitations will strengthen the methodological rigor and broaden the applicability of future research. Expanding empirical inquiry across diverse natural settings, incorporating contextual variables inherent to natural heritage tourism, and adopting longitudinal or experimental designs will not only enhance the robustness of the findings but also deepen understanding of the complex interplay between environmental perceptions, emotional engagement, and responsible behavior in tourism contexts. Such efforts will contribute to advancing environmental psychology and sustainable tourism theory, while supporting more effective management strategies for natural heritage destinations.

## Data Availability

The original contributions presented in the study are included in the article/Supplementary material, further inquiries can be directed to the corresponding author.

## Footnotes

1
https://www.wjx.cn

## Appendix

**Table:**

Questionnaire			
Variables	Items	Question	References
Natural Environment	NE1	The natural scenery of Shennongjia left a deep impression on me.	Zolfani et al. (2015); Chung et al. (2018); Cheng and Monroe (2012)
	NE2	The natural environment of Shennongjia made me feel the greatness of nature.	
	NE3	The landscape of Shennongjia is uniquely beautiful.	
	NE4	The natural environment of Shennongjia makes me feel the grandeur of nature.	
Cultural Environment	CE1	The cultural environment of Shennongjia Forest District has given me a sense of cultural enrichment.	Zolfani et al. (2015); Wu et al. (2020); Zhang Z. et al. (2023)
	CE2	The folk legends of Shennongjia, such as the story of Shennong tasting hundreds of herbs, deeply move me.	
	CE3	The local customs of Shennongjia Forest District are rich in cultural significance (such as the Han Chinese mythological epic The Dark Chronicle, shadow puppetry, and the funeral drumming ritual).	
	CE4	The long history of Shennongjia Forest District is very inspiring.	
Empathy with Nature	EN1	Being in the Shennongjia forest area makes me feel a strong bond with the natural environment.	Tam, 2013; Ballew and Omoto, 2018; Zhou et al., 2025
	EN2	Being in the Shennongjia forest area fosters an internal sense of connection between humans and nature.	
	EN3	Being in the Shennongjia forest area has given me a strong desire to protect the natural environment.	
	EN4	Being in the Shennongjia forest area has given me a deep emotional resonance with everything around me.	
	EN5	Being in the Shennongjia forest area makes me feel like I am part of the natural environment.	
Perceived Escapism	PE1	Visiting Shennongjia makes me feel as if I am in another world.	Orazi et al., 2023; Mirowska and Arsenyan, 2023
	PE2	Being in Shennongjia forest area made me feel far away from reality.	
	PE3	The process of visiting made me forget my surroundings.	
	PE4	The process of visiting made me forget myself and be completely immersed in it.	
Awe	AWE1	The Shennongjia forest area is extraordinary.	Piff et al., 2015; Keltner and Haidt, 2003; Wang and Lu, 2024
	AWE2	The Shennongjia forest area is breathtaking.	
	AWE3	The Shennongjia forest area makes one feel humble.	
	AWE4	The Shennongjia forest area evokes a feeling of humility.	
	AWE5	The Shennongjia forest area exceeded my expectations.	
ERB	ERB1	I am willing to purchase souvenirs to contribute economically to Shennongjia.	Cape Town Declaration (2002); Lin et al. (2022); Manyara and Jones (2007)
	ERB2	I am willing to use paid transportation to provide financial support for Shennongjia.	
	ERB3	I am willing to buy local food specialties to contribute economically to Shennongjia.	
	ERB4	I am willing to donate to Shennongjia to help with environmental protection and facility upgrades.	
NERB	NERB1	I greatly respect the culture of Shennongjia.	Cape Town Declaration (2002); Gao et al. (2017); Manyara and Jones (2007)
	NERB2	I greatly cherish the resources of Shennongjia.	
	NERB3	I am willing to comply with the regulations of Shennongjia.	
	NERB4	I am willing to protect the wildlife and plants of Shennongjia.	
	NERB5	I am willing to protect the scarce natural and cultural resources of Shennongjia.	
